# From perception to inference: Utilization of probabilities as decision weights in children

**DOI:** 10.3758/s13421-020-01127-0

**Published:** 2021-01-15

**Authors:** Tilmann Betsch, Stefanie Lindow, Anne Lehmann, Rachel Stenmans

**Affiliations:** Department of Psychology, University of Erfurt, Nordhaeuser Strasse 63, D-99089 Erfurt, Germany

**Keywords:** Child decision-making, Probabilistic inference, Utilization of decision weights, Perception, Conception

## Abstract

In a probabilistic inference task (three probabilistic cues predict outcomes for two options), we examined decisions from 233 children (5–6 vs. 9–10 years). Contiguity (low vs. high; i.e., position of probabilistic information far vs. close to options) and demand for selectivity (low vs. high; i.e., showing predictions of desired vs. desired and undesired outcomes) were varied as configural aspects of the presentation format. Probability utilization was measured by the frequency of following the predictions of the highest validity cue in choice. High contiguity and low demand for selectivity strongly and moderately increased probability utilization, respectively. Children are influenced by presentation format when using probabilities as decision weights. They benefit from perception-like presentations that present probabilities and options as compounds.

**Supplementary Information:**

The online version contains supplementary material available at 10.3758/s13421-020-01127-0.

What makes a good decision maker? Blaise Pascal, the late-Renaissance mathematician and philosopher, suggested that good decisions mimic the rules of probability. Even in highly consequential choices (e.g., if you are wondering whether to obey the Decalogue or addict yourself to sinful desires; the Wager’s problem; Pascal, 1670), he recommends proceeding like a cold-calculating gambler (i.e., weight the value of consequences by their probabilities and choose the option with the highest expected value). A dice player, for example, may face the following two lotteries: (a) winning 70 Euros if the die shows an even number; (b) winning 90 Euros if the die shows a 1 or 6. Pascal would expect the gambler to bet on lottery (a), because 70 × 1/2 > 90 × 1/3.

Empirical and everyday experiences tell us that individuals do not generally follow this ideal. Sometimes they mistake possibilities for probabilities (e.g., risks as feelings; Loewenstein, Weber, Hsee, & Welch, 2001) or fail to integrate probabilities in a normatively sound fashion (e.g., base-rate neglect; Bar-Hillel & Fischhoff, 1981). Nevertheless, weighting is of paramount importance even in many simple strategies that circumvent the integration of information. Lexicographic strategies (e.g., take-the-best; Gigerenzer & Gaissmaier, 2011), for example, require the individual to detect the most important dimension in a choice situation. Consider, for instance, the standard structure of a Brunswikian probabilistic inference task. There, several cues predict a distal entity (e.g., the future outcomes of options). The cues differ with regard to their validity. In a probabilistic world, the cues’ validities reflect the probabilities that outcomes are predicted correctly.^1^ In an experimental setting, such cues can be advice givers (e.g., testers) who make predictions about the quality of products (Glöckner & Betsch, 2008). If their validities are known and stated, the decision maker who applies a lexicographic strategy should select the advice giver with the highest validity and follow his or her recommendations. In many decision situations, such a simplifying strategy is sufficient and yields comparable accuracy to decisions by weighted information integration (e.g., Payne, Bettman, & Johnson, 1988; see also Gigerenzer & Gaissmaier, 2011). However, differences in validity must still be encoded, and the subsequent focus of attention should be placed on the best cue. Hence, this simplifying strategy still requires weighting (prioritization of a cue), although it relieves the individual from weighted information integration.

At what time in cognitive development are humans able to use probabilities as decision weights?^2^ The work in the field of judgment and decision-making convergences in indicating that the competence to use probabilities as weights emerges rather late in cognitive development. Before the age of 10 years, children tend to neglect probabilities in lottery decisions (Levin & Hart, 2003; Levin, Weller, Pederson, & Harshman, 2007) and probabilistic inference decisions (Betsch & Lang, 2013; Betsch, Lang, Lehmann, & Axmann, 2014; Betsch, Lehmann, Jekel, Lindow, & Glöckner, 2018; Betsch, Lehmann, Lindow, Lang, & Schoemann, 2016; Lang & Betsch, 2018). Moreover, they are reluctant to apply “simple” strategies, such as take-the-best, that shift weighting from integration to search processes (Mata, von Helversen, & Rieskamp, 2011).

These findings are in sharp contrast to post-Piagetian research in developmental psychology. Whereas early studies document probability neglect until secondary school age (11–12 years; e.g., Hoemann & Ross, 1971; Kreitler & Kreitler, 1986; Piaget & Inhelder, 1951), subsequent research suggests astounding abilities to sensitize and utilize probabilistic information even in preschool children (Denison & Xu, 2014; Pasquini, Corriveau, Koenig, & Harris, 2007; Schlottmann, 2001; Schlottmann & Anderson, 1994; but see Girotto, Fontanari, Gonzalez, Vallortigara, & Blaye, 2016, for counterevidence).

## Task-contingent performance: A chance rather than a challenge

Aiming at identifying the origins of conflicting evidence, we face a striking diversity in research paradigms. The presentation of probabilistic information differs. Children sometimes learn probabilities via experiencing frequency distributions (e.g., Pasquini et al., 2007), they are presented with distributions of objects in jars (e.g., Denison & Xu, 2014; Girotto et al., 2016) or different sizes of areas on which a ball can land (e.g., Schlottmann, 2001). The task may require predecisional search for information on a board (e.g., Betsch, Lehmann et al., 2016; Mata et al., 2011) or all relevant information is directly accessible (e.g., Denison & Xu, 2014; Levin et al., 2007; Schlottmann, 2001). Dependent measures also vary substantially, such as patterns of information search (e.g., Betsch, Wünsche, Großkopf, Schröder, & Stenmans, 2018), evaluative judgments (e.g., Schlottmann, 2001), trust in informants (Pasquini et al., 2007), approach behavior (e.g., crawling; Denison & Xu, 2014), or choice between multiple objects (e.g., Betsch, Lehmann, et al., 2018; Levin et al., 2007). These few examples illustrate task diversity.

Presumably, task features may be—at least partly—responsible for the variations of results (see Fiedler, 2011, for a methodological discussion). Some tasks may suit children’s cognitive abilities better than others (see Betsch, Lehmann, Lindow, & Buttelmann, 2020). As a consequence, performance could be contingent on task features. One might assume that tasks differ in their child friendliness (i.e., the ease with which a task can be understood by children). From such a viewpoint, the researcher is responsible for creating tasks that maximize the likelihood that children can reveal their capacities and potentials. Stretching this view to an extreme, one might dismiss studies revealing deficits in children’s performance and blame the researchers for failing to create child-friendly tasks.

On the other hand, mixed evidence offers trajectories for advancing our knowledge. It could be that differences in paradigms and findings reflect an underlying *systematic relation between task dimensions and cognitive development*. Some features may require higher order cognitive abilities so that children can use probabilities as weights, whereas others suit basic intuitive processes that evolve and consolidate very early in development. We consider mixed evidence as a chance rather than a challenge and task features as a potential means to regulate decision behavior in order to better understand how the ability of probability utilization evolves.

## From perception to conception

Wohlwill (1968) came up with an analytical approach to operationally place tasks alongside a continuum based on the extent to which they demand conceptual understanding. Perceptual and conceptual tasks mark its end points. A purely perceptual task can be solved using intuition that immediately arises vis-à-vis the perceptual input. Mastering a conceptual task, in contrast, requires advanced cognitive skills and formal conceptual knowledge. With cognitive development, “there is a decreasing dependency of behavior of information in the immediate stimulus field” (Wohlwill, 1968, p. 472; for a similar view see Schlottmann & Wilkening, 2012). This notion implies that the assessment and generalization of a child’s developmental status is contingent upon the task’s position on the continuum. Solving a perceptual task does not necessarily imply that the child is also capable of solving a conceptual task. Consequently, one must *compare* performance in *different types of tasks* in order to properly assess the level of cognitive development in individuals or a group of a certain age. In the literature on child decision-making, however, researchers commonly assess developmental status within tasks (“their” paradigm). Research camps tend to stick with their particular paradigm and produce strikingly different findings. Not surprisingly, empirical evidence on children’s utilization of probabilities as decision weights is strikingly mixed. There is evidence, for example, that understanding probabilities begins in infancy (Denison & Xu, 2014), whereas others find that children do not become adaptive to probabilistic decision environments until the end of elementary school (Betsch, Lehmann, et al., 2018) or even secondary school (Piaget & Inhelder, 1951).

To advance our knowledge of competency development in humans, researchers should systematically compare the performance of children across tasks. To this end, one needs an analytical framework identifying relevant task dimensions. Wohlwill (1968) put forward an operational, three-dimensional approach, suggesting that redundancy, selectivity, and contiguity are of paramount importance when locating a task on the continuum between perception and conception.

## Task dimensions: Redundancy, selectivity, and contiguity

Broadly speaking, redundancy and selectivity both affect differentiation between the figure and the ground. The more stimuli jointly activate the same impression in the perceiver (increasing redundancy), and the less irrelevant stimuli are present that dilute or distract away from this impression (decreasing demand for selectivity), the closer the task approaches perception. In contrast, in the absence of redundancy and a high demand for selectivity, tasks require conception.

In a groundbreaking study, Bruner, Goodnow, and Austin (1956) gradually varied task attributes on these dimensions. In an object selection task, participants (third-graders, fifth-graders, adults) were presented with sets containing three geometrical figures and were asked to identify the odd ones. Figures varied with regard to shape, color, shading, and size. Over a series of sets, redundancy decreased, whereas the demand for selectivity increased. In most perceptual-like condition, the “odd” object differed on three dimensions (color, size, shading) from the others, yielding high redundancy and a low demand for selectivity. Only one attribute (color) varied between the three objects and thus was irrelevant for identifying the odd object. In this condition, all age groups performed almost equally well and were able to identify the correct object within a narrow time frame. In the most conceptual-like condition, the task was characterized by the absence of redundancy and a high demand for selectivity. Only one attribute (shape) was shared by two objects, whereas none of the other attributes was shared. Accordingly, participants had to detect shape as the only relevant attribute and consequently select the object that differed in shape from the others. In this condition, error rate differed strongly between children and adults. Moreover, even in adults, mean reaction times doubled in comparison to the perceptual task condition.

The third dimension in Wohlwill’s framework is contiguity, a dimension well known to affect virtually all aspects of cognition and behavior. In perception, causal attribution, and learning, just to mention some domains, spatial and temporal distance of stimuli heavily affect information processing in the individual. For instance, the distance between central and contextual stimuli is responsible for a number of perceptual illusions (e.g., Attneave, 1954)—stimuli that occur at the same time or in rapid succession are likely to be associated in memory (e.g., Hebb, 1949) or used as candidates for causal attribution (e.g., Heider & Simmel, 1944). The higher the contiguity of relevant stimuli or stimulus features, the more likely it is that the task can be solved without higher levels of conception.

For illustration, consider two tasks from research on probability utilization in children. They produced strikingly different results in children’s utilization of probabilities. Schlottmann (2001) demonstrates that even preschoolers can integrate probabilities and values in a multiplicative-like fashion, as predicted by utility theory. In her task, she visualized the probability and value of outcomes in the following manner (see also Schlottmann & Wilkening, 2012, p. 62). A marble was shaken in a tube with two clusters of coloured segments (e.g., blue, yellow). Above each cluster, the potential gain was depicted (crayons). Value was manipulated by varying the number of crayons above each cluster (e.g., six for blue, one for yellow). Probability (e.g., 80% chance of winning if the marble stops in the blue cluster) was manipulated by varying the number of segments in a cluster (e.g., four segments in the blue, one segment in the yellow cluster). In the marble tube task, spatial contiguity between probability and value is high. The crayons are depicted directly above the right and left cluster in the tube. The blue and the yellow clusters represent the two potential outcomes of the task. As such, the outcomes contain all relevant information in a contiguous arrangement—values (number of crayons) and probability (size/number of segments).

**Fig. 1:**
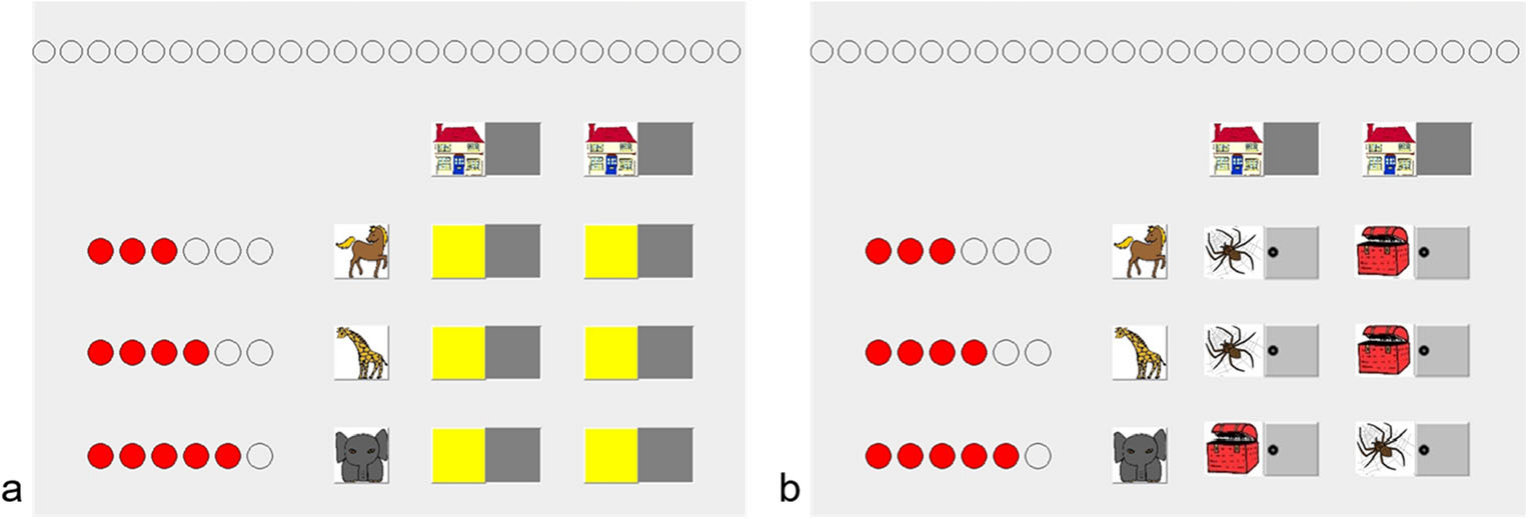
Screenshots from Mousekids (Betsch, Lehmann, et al., 2016). **a** Standard version. Individuals can search the predictions of three animals (cues) that are hidden behind closed matrix cells. By touching a matrix cell, the icon of a treasure or spider appears and indicates what the animal thinks is contained in the house (outcome predictions for options). The number of “smart circles” at the margin of the information board indicate the cue validities (i.e., the probabilities that the animals’ predictions are correct; i.e., 3 out of 6; 4 out of 6; 5 out of 6). **b** Open-board version of the game. The animals’ predictions are presented uncovered without the need of active information search (Betsch et al., 2014; Betsch, Lehmann, et al., 2018)

Betsch and colleagues (2014; Betsch, Lehmann, et al., 2018; Betsch, Lehmann, et al., 2016) used an information board approach to study probabilistic inference decisions in children. The task contains several pieces of relevant information that appear at different locations on the information board and must be combined (see Fig. 1 for the computerized version Mousekids). Specifically, the presentation comprises options (houses), cues (animals), outcomes predicted by cues (whether a house contains a treasure or a spider), and the cue validities (probability that a cue makes correct predictions, represented by “smart circles”). Notably, cue validities are spatially dissociated from the options because these types of information appear at the margins of the information board matrix. In contrast to Schlottman’s results, Betsch and colleagues (2014; Betsch, Lehmann, et al., 2018; Betsch, Lehmann, et al., 2016) consistently found that preschoolers (around 5–6 years old) do not utilize probabilistic information. Specifically, they did not systematically prioritize the predictions of the cue with the highest validity (HVC; i.e., they frequently preferred the option that was not recommended by the HVC). And still two thirds of elementary schoolers (9–10 years old) also neglected probabilities in their decisions, as evidenced by choices in opposition to the HVC’s recommendation. This overall probability neglect is reported for the standard information board (see Fig. 1a), where information must be searched behind closed matrix cells (Betsch et al., 2014; Betsch, Lehmann, et al., 2016), as well as for the open board (see Fig. 1b), where all information is presented uncovered (Betsch et al., 2014; Betsch, Lehmann, et al., 2018). Both paradigms, the marble tube task and the information board paradigm, involve similar information that must be processed. That is, several values and probabilities (presented as magnitudes) describe two options. However, according to Wohlwill’s (1968) framework, the marble tube task is closer to perception than the information board paradigm due its spatial contiguity (see Betsch, Lehmann, et al. 2018, for a discussion).

## Research approach: Varying task dimensions within the same paradigm

**Fig. 2:**
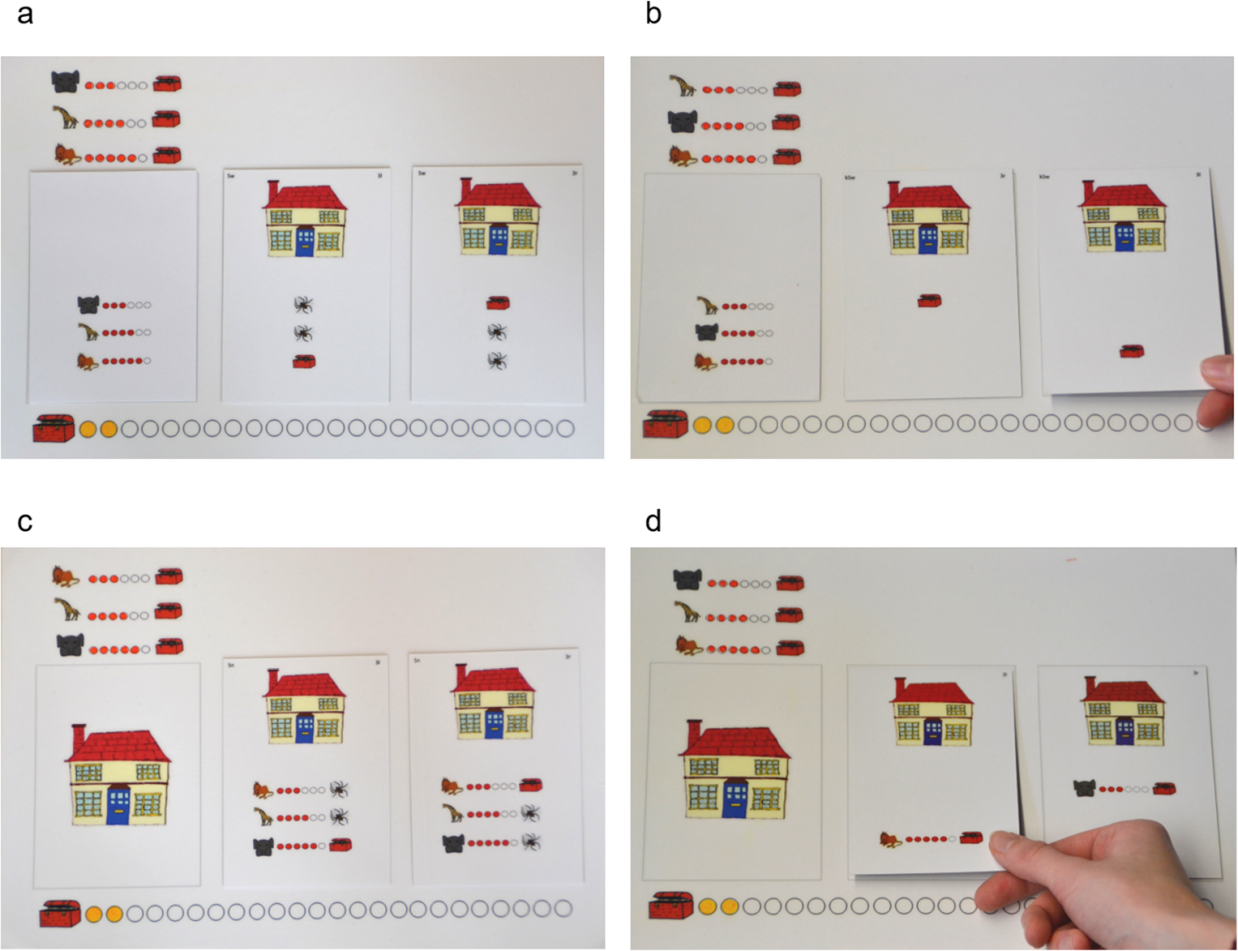
Mousecards. **a–d** The four different experimental conditions. **a** The replication condition (see Fig. 1b). In this condition, the cards contain two types of predictions (treasures, spiders). The cues (animals) with their cue validities (“smart circles”, reflecting the probability that a cue’s prediction will be correct) appear at the margins. As such, selectivity and contiguity are not altered in comparison to the former paradigm (Mousekids; Betsch et al., 2014, Betsch, Lehmann, et al., 2016; Lang & Betsch, 2018). Participants make their choice by drawing one of the cards from the game board and turning it upside down to inspect the outcome. If the house contains a treasure, participants color in a “treasure point” at the bottom of the game board

Due to a plethora of potential confounds, it is difficult to draw valid conclusions from comparing tasks from different paradigms. Therefore, a promising approach might be to systematically vary task dimensions within the same paradigm. In this study, we adopted the presentation format of Betsch and colleagues’ information-board approach. We varied contiguity and selectivity as between-subjects factors and used a presentation format similar to the open-board condition (Betsch et al., 2014). All information is presented uncovered, thus eliminating the need for active information search (opening cells in the matrix). In deviation from prior presentation formats, we presented the task on cards in order to vary configural aspects (contiguity and demand for selectivity). Figure 2 shows the replication condition (see Fig. 2a) and all of the new experimental conditions in which contiguity is increased and/or demand for selectivity is decreased. By removing predictions of the undesired outcome—spiders—the matrix only contains the relevant predictions of the cues (i.e., the treasures). Without spiders, the amount of displayed information substantially decreased, as did the demand for selectivity. Contiguity was increased by moving probabilistic information (the “smart circles”) from the margins onto the cards. As a consequence, the spatial distance between the picture of the house, the predicted treasures, and their probabilities was minimized. All these features appear together on a single card, thus forming a perceptual unity of the eligible option. Importantly, the structure of the task is preserved. Only spatial relations are altered.

## Hypotheses

We examined the utilization of probabilities in choice in probabilistic inference tasks. In our noncompensatory environment,^3^ it is normatively appropriate to prioritize the predictions of the HVC (see explanation of research paradigm in the Methods section for further explanation). The utilization of probabilities should therefore manifest itself in choices that are consistent with the HVC’s predictions.

We predict two main effects for contiguity and selectivity:

- H1: In the conditions with high contiguity (see Fig. 2c–d), the frequency of choices that are consistent with the predictions of the HVC increases compared with the conditions with low contiguity (see Fig. 2a–b).
- H2: In the conditions with a low demand for selectivity (see Fig. 2b, d), the frequency of choices that are consistent with the predictions of the HVC increases compared with the conditions with a high demand for selectivity (see Fig. 2a, c).

We also expect that the previously found effect for age (Betsch et al., 2014; Betsch, Lehmann, et al., 2018; Betsch, Lehmann, et al., 2016) will replicate in the altered paradigm:

- H3: The frequency of choices that are consistent with the predictions of the HVC is higher in elementary schoolers than in preschoolers.

## Method

This research has been approved by the research ethics board of the University of Erfurt (project title BE 2012/11-2).

### Participants and design

The selection of age groups and sample size (*n* > 25 per condition) is consistent with prior research (Betsch et al., 2014; Betsch, Lehmann, et al., 2016). Sample size allows for detecting main effects of a medium effect size in an analysis of variance (ANOVA) with α = .05, 1 − β > .85 according to a power analysis conducted with G*Power (Faul, Erdfelder, Lang, & Buchner, 2007). We studied two different age groups: 6-year-olds (*n* = 117; 50.4% female; age: *Mdn* = 74 months, *M* = 73.33, *SD* = 5.28) and 9-year-olds (*n* = 116; 60% female; age: *Mdn* = 109 months, *M* = 108.53, *SD* = 6.15). Within each age group, participants were randomly assigned to one of four conditions resulting from a 2 (contiguity high vs. low) × 2 (demand for selectivity high vs. low) design. Children (all native German speakers) were recruited in elementary schools and daycare centers located in middle-class areas of a moderately large city in central Germany. Parents had previously provided consent for their children to participate in child development research. Additional children (*n* = 7) were tested but excluded from analyses because they did not pass the manipulation check (i.e., they rated one of the low validity cues to be smarter than the HVC after the learning sessions; see below, *n* = 4) or they did not finish the test session (*n* = 3).

### Research paradigm: Mousecards

We developed a card game version in accordance with the information board environment used in prior research (Mousekids; Betsch, Lehmann, et al., 2018; Betsch, Lehmann, et al., 2016). Participants were on a treasure hunt and repeatedly chose between two houses (i.e., two choice options). They received a “treasure point” each time they chose the house containing a treasure. Participants had to collect as many “treasure points” as possible. Three cues (animals) served as advice givers to help participants in their choices. For each choice, the animals predicted in which house the treasure might be located. Importantly, the animals differed in their validity (i.e., in the probability that their predictions were correct). Most predictions of the HVC were correct (*p* = .83), whereas the lower validity cues were less frequently correct (*p* = .67) or uninformative (*p* = .50; see Betsch et al., 2014).

The game consisted of two parts: a learning session and a test session. In the initial learning session, participants learned the validities of the cues. Specifically, they observed the performance of the animals in six learning trials per cue. For each correct prediction an animal made, it received a “smart circle.” At the end of the learning session, cue validities were graphically represented by the number of “smart circles” the animal had earned (3 out of 6, 4 out of 6, 5 out of 6). In the subsequent test session, participants made their choices with the help of the three animals in 24 target trials. Prior to those, they worked on two practice trials.

**Fig. 3:**
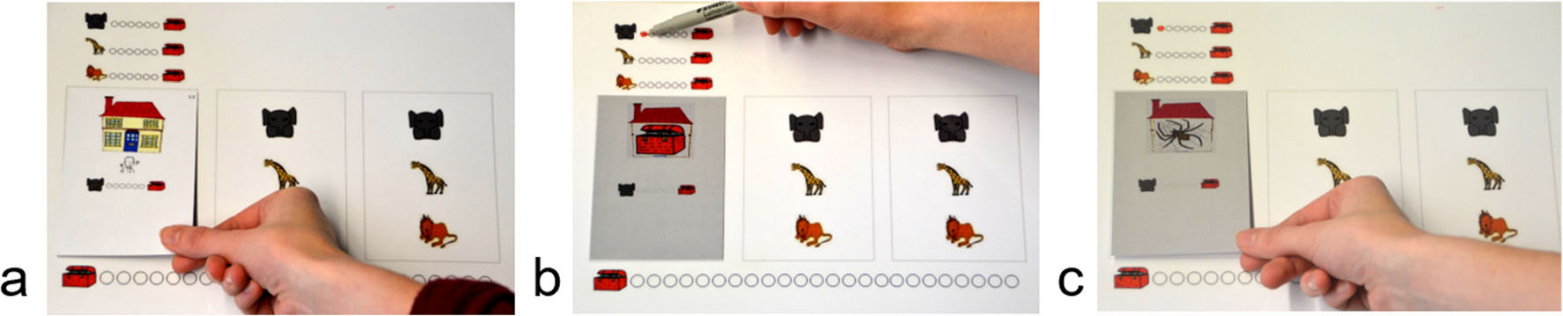
Game board of Mousecards and two example cards of the learning session. **a** The animals’ prediction on the front side of a learning card. **b** An example of a backside (grey background) in which the house actually contains a treasure. **c** An example of a backside in which the house contains a spider. The two areas on the right sides of the game board were used only in the test session and not in the learning session

The learning session consisted of a stack of 18 learning cards (six per cue). As shown in Fig. 3, the back of the learning cards displayed one of the three animals underneath a house with a questioning person. On the front of each learning card (white background; see Fig. 3a), the animal predicted a treasure in the house. When turned upside down (grey background; see Fig. 3b–c), the card showed what was actually contained in the house: either a treasure or a spider. When a treasure was shown, the animals’ prediction was correct. In this case, the animal received a “smart circle.” which participants colored in on the upper left part of the game board (see Fig. 3b).

The test session employed two stacks of 26 test cards, one stack for each option, placed on the two right areas on the game board. On the top, the test cards each showed a house (see Fig. 2). Below the houses, the predictions of the animals were shown. The display of these predictions varied between experimental conditions. Specifically, in the two conditions with high contiguity, the test cards showed all decision-related information (i.e., both the predictions together with the animals and their “smart circles”; see Fig. 2c–d). In contrast, in the conditions with low contiguity, the test cards only displayed the cues’ predictions. A separate card, positioned at the margin, displayed the animals with their “smart circles” (see Fig. 2a–b). In the two conditions with high demand for selectivity, the cues also made predictions about the undesired outcome (spider; see Fig. 2a–c). To achieve a low demand for selectivity, we removed the spiders from the test cards. As shown in Fig. 2b and d, the test cards only showed the relevant predictions (i.e., the treasures).

The front side looked the same as the respective backside, with the exception that it was grey shaded and that the actual outcome (treasure or spider) appeared in the house to indicate its content (participants had already been familiarized with this presentation during the learning phase; see Fig. 3).

**Fig. 4:**
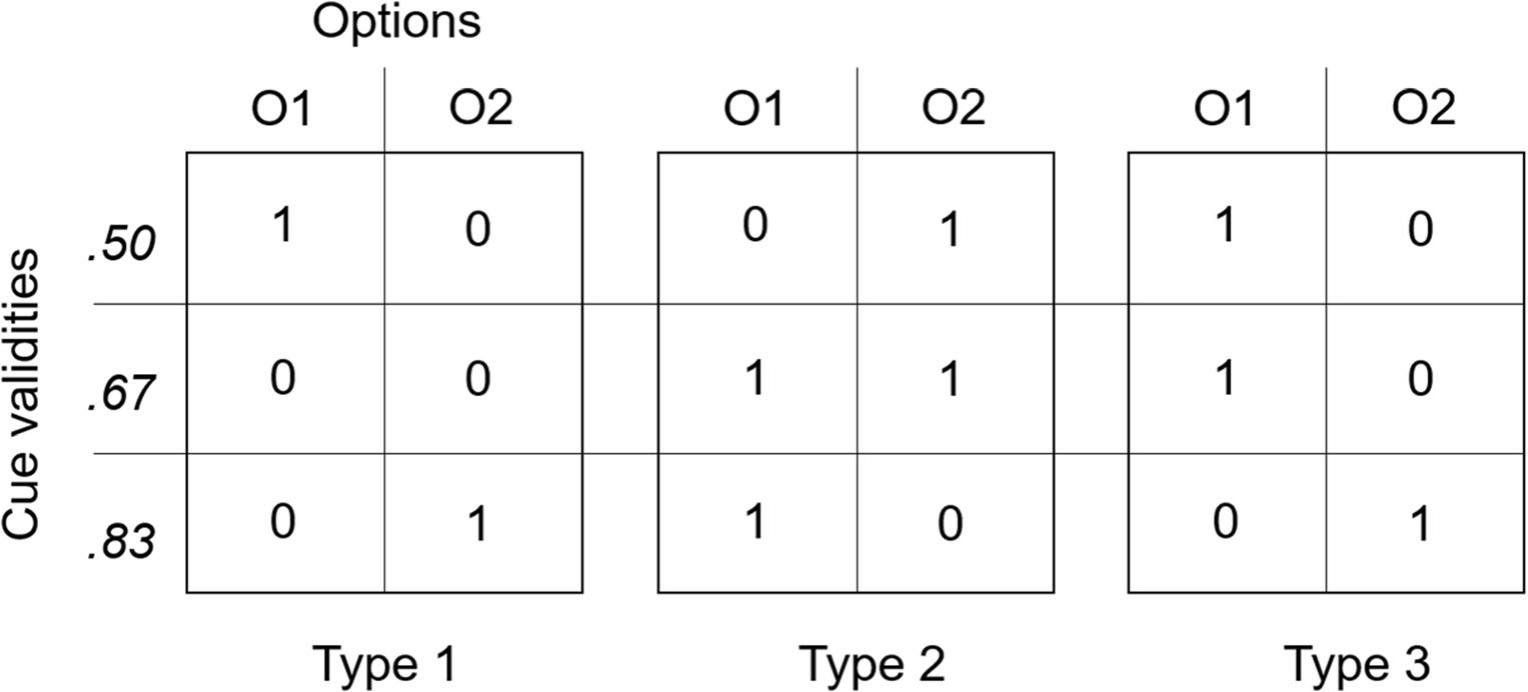
Types of prediction patterns. Each pattern was used four times in the depicted manner and four times in a mirrored version. In the mirrored version of the Type 1 pattern, for example, the low validity cue predicts a gain (“treasure”) for Option 2, while the HVC predicts a gain for Option 1 (adapted from Betsch et al., 2014)

Following Betsch et al. (2014), we employed three types of prediction patterns (see Fig. 4). In the game, two versions of the patterns appeared equally often, either as shown in Fig. 4 or in a mirrored version. In the first two types, the HVC (*p* = .83) predicted a different option than the cue with the lowest validity (*p* = .50), whereas the cue in the middle (*p* = .67) was indifferent—either not predicting a treasure in any house (Type 1) or predicting a treasure in both houses (Type 2). In Type 3, the HVC contradicted the predictions of the two remaining cues. Note that regardless of pattern type, all decision tasks were noncompensatory because the joint prediction of the two cues with lower validity cannot compensate for a prediction of the HVC. The cue with the lowest validity (*p* = .50) is normatively uninformative (e.g., Lee, 2016; see Footnote 3). If participants consider cue validities, they should always follow the HVC and choose the option predicted by that animal.

Payoffs were arranged in such a fashion that marginal probabilities were identical for the two options (i.e., the probability that the left or the right house contains a treasure was *p* = .50). Thus, feedback did not differentially reinforce right or left choices. Moreover, differences in cue validities were replicated with respect to the hit and false-alarm rates of the cues’ predictions. Specifically, payoffs reinforced the low validity cue in 50%, the medium validity cue in approximately 62%, and the HVC in 83% of the trials. To achieve this, both houses contained treasures in eight trials. Note that a perfect replication of the cues’ validities in the payoffs is not possible due to arithmetic constraints.

### Procedure

The experimenter met the child in a quiet room at the primary school or daycare center. As in Mousekids (Betsch, Lehmann, et al., 2016), in Mousecards the experimenter first explained that the purpose of the game is to find treasures. Then, the experimenter introduced the three animals (a lion, an elephant and a giraffe) that would play the game together with the child and help the child to find treasures. The child stated which of the three animals was the favorite animal. Accordingly, the experimenter chose the deck of cards with the child’s favorite animal on top (i.e., the lowest validity cue).

The purpose of the subsequent learning session was to demonstrate how smart the animals were. The experimenter told the child: “Do you see the house up there? Maybe there is a treasure in the house, and maybe not. The animals will tell you whether there is a treasure hidden there or not. But the animals are not always right. Therefore, we are going to check how often they are right.” Subsequently, each animal made six predictions. For each prediction, the experimenter placed a learning card on the game board that showed that the animal predicted a treasure in the house (see Fig. 3a). Then, the experimenter turned the learning card upside down. When the back showed a correct prediction, the child was instructed to award a “smart circle” to the animal by coloring in one of the circles next to the animal (see Fig. 3b). When the back of the learning card showed a spider, the animal did not receive a “smart circle.” because it had made an incorrect prediction (see Fig. 3c). After six learning trials, the experimenter pointed out, “Now we know how smart the animal is,” and summarized the number of “smart circles” gained by the animal (e.g., “the animal gained 3 out of 6 smart circles” if *p* = .50). After having finished the learning session for all three animals, the experimenter asked the child which of the animals was the smartest. This question served as the *manipulation check* for learning the cue validities.

Then, the experimenter removed the learning cards from the game board and proceeded with the cards of the test session. Depending on the experimental condition, the experimenter either placed only the two cards with the predictions on the two houses (Fig. 2c–d) or these two cards together with an additional card showing the three animals with their “smart circles” (Fig. 2a–b) on the game board. The experimenter then explained the goal of the game (“you have to find as many treasures as possible in order to buy more prizes afterwards”), payoffs (treasure can be in one of the houses, in both, or in none), actions (choosing a house and coloring treasure points after success), and cue predictions. In the experimental conditions with a high demand for selectivity, the experimenter explained that the animals predict either a treasure or spider. In contrast, in the experimental conditions with low demand for selectivity, the experimenter explained: “In this game, you will be shown when the animals expect a treasure in the house. When the animals say nothing, it means that they think that a spider is in the house.”

Preceding the test session, the child played two warm-up trials, which did not count towards overall performance. In the first warm-up trial, the experimenter verbalized the prediction of the animals before the child chose a house. In the second warm-up trial, it was the child’s turn to explain the prediction of the animals. This was used to check whether the child understood the game board. If the child’s explanation was incorrect or incomplete, the experimenter corrected it. In the subsequent 24 trials of the test session, the experimenter asked the child to make a choice without further verbalization of the predictions. If the chosen house contained a treasure, the child colored in a treasure point at the bottom of the game board (see Fig. 2). If it contained a spider, the child earned no treasure point.

After completing the test session, the experimenter asked the child to state which of the animals was the smartest and which was the second smartest with respect to predicting where treasures were hidden. Then, the child could trade the treasure points for actual prizes. Performance-contingent awarding ensured that children were motivated to make accurate decisions. Moreover, every child received a personalized certificate and was thanked for participating.

## Results

### Hypotheses testing

**Fig. 5:**
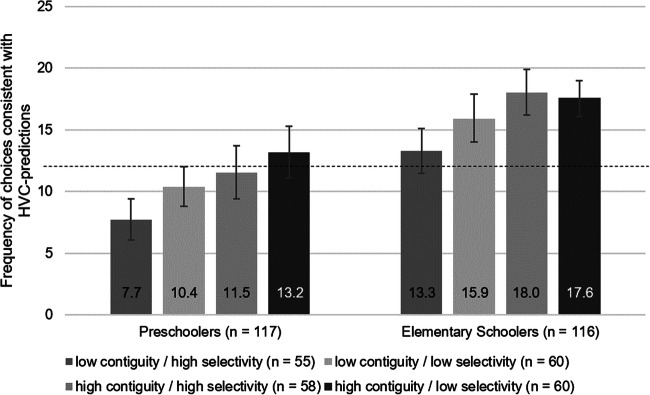
Mean frequencies of choices that are consistent with predictions of the HVC for the age groups and the four variations of the presentation format. Error bars indicate 95% CIs. Low contiguity / high selectivity = replication condition with low contiguity (cues are placed at the margins) and a high demand for selectivity (spiders, i.e., the predictions for the nondesired outcomes, are also presented). The test session had 24 trials. The dashed line indicates chance performance.

Figure 5 shows that the frequency of choices that are consistent with the predictions of the HVC differs markedly between experimental conditions and age groups. In both age groups, the lowest mean values show up in the replication condition, in which contiguity was low (cues appear at the margins) and demand for selectivity was high (information about the undesired outcome also appear). We conducted a full-factorial ANOVA, with contiguity, selectivity, and age as independent variables. Corroborating Hypothesis 1, the frequency of choices consistent with the HVC’s predictions increased from the low to the high contiguity conditions, *F*(1, 225) = 25.911, *p* < .01, η_p_^2^ = .103. A similar but weaker main effect was found for selectivity, *F*(1, 225) = 6.475, *p* = .012, η_p_^2^ = .028. In line with Hypothesis 2, the frequency of choices consistent with the HVC’s predictions is larger in the conditions with a low demand for selectivity compared with the conditions with a high demand. No interaction effects with age were found, indicating that the influence of configural aspects of the presentation format works the same in both age groups (all *F*s < 1). Finally, we found a strong effect for age, *F*(1, 225) = 74.778, *p* < .01, η_p_^2^ = .249. As predicted in Hypothesis 3, elementary schoolers’ choices were more frequently consistent with the HVC’s predictions than choices in preschoolers (there were no other significant effects), Effect Selectivity × Contiguity interaction, *F*(1, 225) = 2.568, *p* = .11, η_p_^2^ = .011; Effect Age × Selectivity × Contiguity interaction, *F* < 1.

### Exploratory analyses

As in prior studies, we varied prediction patterns of the cues (see Fig. 4). In an exploratory step, we analyzed whether contiguity and selectivity influenced choice behavior differently in the three pattern types. We considered the frequency of choices that are consistent with the predictions of the HVC pattern wise, yielding three dependent measures that could range from 0 to 8 because participants encountered each pattern eight times (for descriptive statistics, see Table 1). We subjected these three dependent variables to a repeated-measures ANOVA, with contiguity, selectivity, and age group as between-subjects factors. Contiguity and selectivity did not interact with pattern type (all *F*s < 1.7). This means that contiguity and selectivity did not differentially effect choice behavior and are thus not dependent on specific constellations of predictions.

**Table 1:** Mean frequencies of choices that are consistent with the predictions of the HVC depending on experimental condition

	Preschoolers				Elementary schoolers			
	Low contig / high select	Low contig / low select	High contig / high select	High contig / low select	Low contig / high select	Low contig / low select	High contig / high select	High contig / low select
Type 1	2.48 (1.91)	3.50 (1.71)	4.07 (2.12)	4.47 (2.13)	4.71 (1.92)	5.67 (2.01)	6.21 (1.91)	6.67 (1.63)
Type 2	2.70 (1.90)	3.53 (2.00)	4.00 (2.17)	4.60 (2.37)	4.89 (2.22)	5.93 (1.96)	6.36 (1.79)	6.57 (1.63)
Type 3	2.56 (1.76)	3.37 (2.17)	3.50 (2.32)	4.13 (2.24)	3.71 (2.02)	4.33 (2.47)	5.46 (2.44)	4.33 (2.25)
Half 1	3.90 (2.09)	4.73 (2.24)	5.30 (2.61)	6.50 (2.86)	6.61 (2.75)	7.80 (2.73)	8.57 (2.82)	9.20 (2.00)
Half 2	3.89 (2.82)	5.67 (2.73)	6.23 (3.90)	6.70 (3.41)	6.71 (2.62)	8.13 (3.16)	9.46 (3.06)	8.37 (2.33)
	*t*(26) = .07 p* = .95 *d* = .01	*t*(29) = 1.94 *p* = .06 *d* = .35	*t*(29) = 1.60 *p* = .12 *d* = .29	*t*(29) = .36 *p* = .72 *d* = .06	*t*(27) = .21 *p* = .83 *d* = .04	*t*(29) = .67 *p* = .51 *d* = .12	*t*(27) = 1.38 *p* = .18 *d* = .26	*t*(29) = −2.59 *p* = .02 *d* = .47

*Note.* Low contig / high select = replication condition with low contiguity (cues are placed at the margins) and high demand for selectivity (spiders, i.e., the predictions for the nondesired outcomes are also presented). Standard deviations in parentheses. Paired-sample *t* tests for Half 1 (i.e., Trials 1 to 12) − Half 2 (i.e., Trials 13 to 24) comparisons. *Bonferonni corrected α = .006

In the repeated-measures part of the analysis, we only obtained two effects, one main effect for pattern type, *F*(2, 450) = 22.864, *p* < .01, η_p_^2^ = .092, and a two-way Pattern Type × Age Group interaction, *F*(2, 450) = 10.289, *p* < .01, η_p_^2^ = .044 (all *F*s < 1.7). As is evident from Table 1, the lowest mean frequency of choices that are consistent with the predictions of the HVC was found in the third pattern, in which the HVC contradicted the predictions of the remaining cues. This difference caused the main effect for the repeated-measures factor. The interaction effect reflects the finding that choice frequencies of the two age groups approached each other in the third pattern replicating prior findings (e.g., Betsch et al., 2014). Due to the additivity of the three dependent measures, the between-subjects effects reported in the ANOVA conducted for hypothesis testing were perfectly replicated; thus, we refrain from reporting them here.

For exploration purposes, we attempted to classify strategies. We hasten to add that this research was not designed for sophisticated strategy classification (see Betsch, Lehmann, et al. 2018, for strategy classification in children). As such, the following results should be considered with caution. We classified participants according to their choice behavior using an outcome-based strategy classification method (Bröder & Schiffer, 2003). We followed the procedure from prior work and classified the same set of strategies (see Betsch, Lehmann, et al., 2020). For each individual, we determined the likelihood of the observed choices under each of the considered strategies (choice model), assuming that strategies were applied with some error (error rate less than .05). Individuals were classified to a choice model if their choices fitted the model’s predictions perfectly or if the likelihood for the classified strategy was higher than for any other strategy. Otherwise, the classification was considered unreliable, and participants remained unclassified. Individuals with equal likelihoods for two strategies also remained unclassified. We assumed a uniform probability distribution of making errors across all decisions within individuals, but variation between individuals. Table 2 shows the classification results.

**Table 2:** Results of exploratory strategy classification

	Preschooler								Elementary schooler							
	Low contig / high select		Low contig / low select		High contig / high select		High contig / low select		Low contig / high select		Low contig / low select		High contig / high select		High contig / low select	
	*n*	%	*n*	%	*n*	%	*n*	%	*n*	%	*n*	%	*n*	%	*n*	%
TTB	3	11.1	2	6.7	8	26.7	8	26.7	9	32.1	15	50.0	18	64.3	18	60.0
EQW	7	25.9	7	23.3	3	10.0	1	3.3	4	14.3	6	20.0	2	7.1	7	23.3
TTF	12	44.4	11	36.7	13	43.3	14	46.7	5	17.9	5	16.7	6	21.4	1	3.3
SW	5	18.5	10	33.3	6	20.0	7	23.3	10	35.7	4	13.3	2	7.1	3	10.0
Unclass.	0	0	0	0	0	0	0	0	0	0	0	0	0	0	1	3.3
Total	27		30		30		30		28		30		28		30	

*Note*. TTB = Take-The-Best; EQW = Equal Weight; TTF = Take-The-First; SW = switching between options; Unclass. = individuals who could not be classified. We also considered random guessing in the strategy classification, but no participant was classified to behave randomly

The first strategy is take-the-best (TTB; Gigerenzer & Goldstein, 1996), a lexicographic strategy. TTB only considers the predictions of the HVC and follows its positive prediction (treasure). The second strategy, equal weight (EQW; Payne et al., 1988), tallies positive predictions while ignoring differences in probabilities. The third strategy, take-the-first (TTF), follows the predictions of the cue at the top of the board, which has the lowest validity. Application of TTF might reflect the individual’s reading habits and a tendency for selective inspection of the information board (see Betsch, Wünsche, Großkopf, Schröder, & Stenmans, 2018). Fourth, we checked for individuals who tended to switch between options (SW; see Lang & Betsch, 2018). Fifth, we counted individuals who could not be classified.

The highest rates of TTB users are obtained under high contiguity, in preschoolers and elementary schoolers, although the rate is lower in the former than in the later. This finding substantiates the results from hypothesis testing above. The second important observation is that the rates for the maladaptive strategies, TTF and SW, are much higher in preschoolers than in elementary schoolers. Roughly 16% of individuals from both age groups are classified as applying the probabilistic EQW strategy, which relies on tallying the number of the cues’ positive predictions (treasure). Interestingly, the overwhelming majority of children can be classified when we allow for an error rate of less than .05. This finding suggests that our model space covers the toolbox of strategies that children use.

Moreover, classification results allow us to rule out some alternative interpretations of the data. Assume, for example, that children have relied on the overall impression of positive information closely associated with the option. In the conditions with high contiguity, the option cards contain not only the symbols of treasures in red color but also the smart circles of the cues depicted in red color. Thus, the high contiguity manipulation might have invited application of a mental magnitude strategy that makes choices by the magnitude of red color or the sum of treasure symbols and smart points.^4^ A mental magnitude strategy, however, would not discriminate options in the conditions with high selectivity in two thirds of the choices. Due to pattern construction, Type 1 and Type 2 patterns provide the magnitude of red in the conditions with high contiguity (the sum of smart circles and treasure symbols is 13 for Type 1 and 14 for Type 2 in both options). Accordingly, the application of the mental magnitude strategy would lead to indecisiveness in two thirds of the choices. In the conditions with low selectivity, however, a mental magnitude strategy discriminates and would lead to the same choices as if the individual would have applied a TTB strategy. If the manipulation of high contiguity (i.e., moving cues and smart points into the representation of the option) would have fostered the application of a mental magnitude strategy, the rate of individuals classified as TTB users should increase in the conditions with low selectivity and decrease in the conditions with high selectivity within those that were presented with high contiguity. Moreover, the rate of unclassified individuals or those who reside with nonanalytic strategies such SW or TTF should increase in the condition with high selectivity (and high contiguity).

The information shown in Table 2 does not indicate such an effect for selectivity within the conditions with high contiguity. The rate of TTB users is equally high in preschoolers (26.7%) and quite similar in elementary schoolers (60% to 64.3%). Only one child was unclassified in these conditions, and rates of users of nonanalytic strategies do not vary in the direction as expected by a mental magnitude strategy. These findings speak against an alternative interpretation in terms of a mental magnitude approach.

**Fig. 6:**
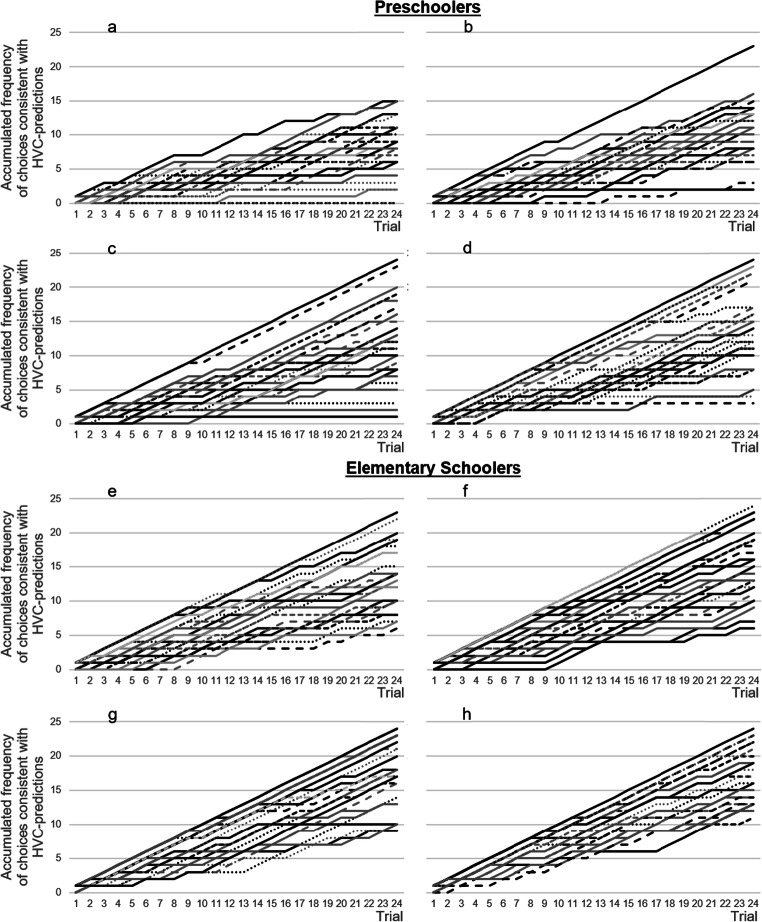
Individual performance over the 24 trials (horizontal axis) in all conditions. The vertical axis shows the accumulated number of choices consistent with the predictions of the HVC separately for each participant.. **a** Low contiguity/high selectivity. **b** Low contiguity/low selectivity. **c** High contiguity/high selectivity. **d** High contiguity/low selectivity. **e** Low contiguity/high selectivity. **f** Low contiguity/low selectivity. **g** High contiguity/high selectivity. **h** High contiguity/low selectivity

**Fig. 7:**
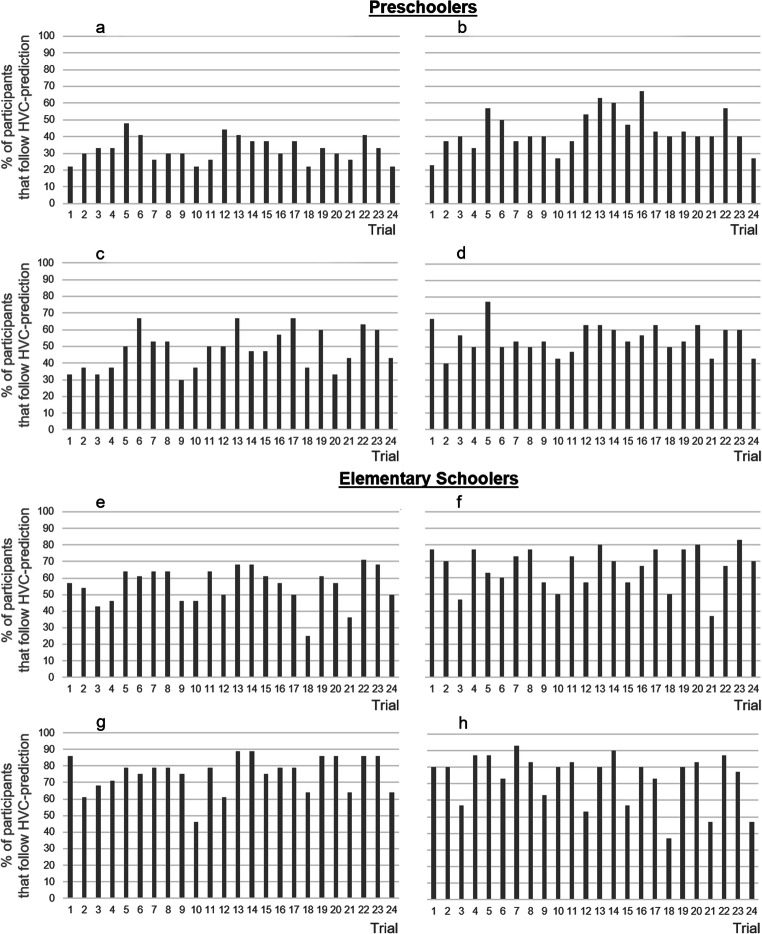
Rate of participants that follow the prediction of the HVC in each of the 24 trials in all conditions. **a** Low contiguity/high selectivity. **b** Low contiguity/low selectivity. **c** High contiguity/high selectivity. **d** High contiguity/low selectivity. **e** Low contiguity/high selectivity. **f** Low contiguity/low selectivity. **g** High contiguity/high selectivity. **h** High contiguity/low selectivity

To investigate potential influences of feedback learning or motivational losses, we analyzed children’s responses across test trials. As in prior studies (e.g., Betsch et al., 2014), there were only slight changes in performance between the first and second half of trials (see Table 1). There was a slight tendency towards improvement. Only in one of eight conditions did performance decrease (elementary schoolers, condition with high contiguity and low selectivity). Additionally, the inspection of the individual performance curves across the 24 trials (see Fig. 6) suggests a linear trend towards an increase in accumulated HVC-consistent choices (*y*-axis) over trials (*x*-axis), additionally suggesting that motivation did not decrease during the experiment. Figure 7 shows the rate of individuals (*y*-axis) that followed the HVC on each of the 24 trials (*x*-axis). If individuals profited from feedback, one would expect an increase over trials. However, the average rate of HVC followers obviously remains quite stable. Altogether, we can rule out the possibility that results are biased by participants’ loss of motivation. Still, participants did not systematically profit from feedback after choice. Recall that feedback reinforced the validities of the cues. Such a feedback structure is necessary, but is not a sufficient condition to enhance learning in the proposed direction. Bröder and colleagues (Bröder, Glöckner, Betsch, Link, & Ettlin, 2013) varied feedback in multiattribute decisions so that it reinforced either option or strategy routinization. The authors showed that the direction of learning varies strongly dependent on subtle features of the presentation. According to the attention-gated learning approaches (e.g., Roelfsema & van Ooyen, 2005), the distribution of attention determines individual’s learning from feedback. In our tasks, there are at least three categories that individuals may attend to when they encode the feedback. Accordingly, finding a treasure may be associated with the option, the cue, or a certain strategy. Note that the environment may foster the association between feedback and the option. The option is represented by a card. The feedback appears on the option if the individual turns over the card. This presentation format may have obstructed cue reinforcement and hence might explain why feedback had a null effect on performance.

## Discussion

At what age are humans capable of using probabilities as decision weights? Conflicting evidence in the literature makes it difficult to answer this question. Aiming at identifying origins of variance in results, we studied the effects of configural aspects of presentation format in a probabilistic inference task. As a theoretical background, we draw on a model put forward by Wohlwill (1968). According to this approach, tasks are characterized by the extent to which they demand conceptual understanding. A purely perceptual task can be solved upon an intuition that immediately arises vis-à-vis the perceptual input. Mastering a conceptual task, in contrast, requires advanced cognitive skills and formal conceptual knowledge. Wohlwill defined three key dimensions that determine task position on a scale with perception and conception at its end points: contiguity, selectivity, and redundancy. In this study, we orthogonally varied two of these dimensions: contiguity and selectivity. In support of our hypotheses, an increase in contiguity and a decrease in the demand for selectivity reduced probability neglect in children. These findings highlight the importance of task characteristics. Children up to 10 years of age are influenced by presentation format when it comes to using probabilities as weights in decision-making.

### Why presentation format matters

Children benefitted from a lower demand for selectivity that was achieved by removing predictions of the undesired outcome. As such, this manipulation reduced the amount of information in the presentation, and, hence, reduced distraction by irrelevant information. A finding that may not be overly surprising if one assumes that (younger) children are likely to fail to suppress distracting information due to immature executive control (Diamond, 2013).

The effect for contiguity, however, is striking. Shifting cues’ positions strongly impacted decisions. We believe that this is not only an effect of changing distances. In the high contiguity condition, cues together with their predictions were included in the presentation of the option itself (the card). As such, the cards formed *compounds* integrating several pieces of information in the representation of the option.

Compound representations of options is a natural format. The following incident was reported by the daughter of one of the authors. Hannah and Kati are soccer enthusiasts. They plan to put together a soccer team at school. Recently, a new girl joined the class. Yesterday, she wore a number 10 jersey from the local soccer club. Although they are not friends yet, Hannah suggests asking her to join their team. “Yup,” says Kati, “great idea. She’ll surely stir up the midfield.” Kati’s judgment reveals a great deal of underlying inferences. She takes the jersey as a cue for expertise in soccer. Presumably, she assumed that the girl actually plays in a junior team of the local club. Because of the prestigious number 10 (associated with playmakers), Kati expects the girl to perform excellently on the soccer field. This is a probabilistic inference par excellence*.* Importantly, however, the probabilistic cue (the jersey) is an integral part of the representation of the “option” (i.e., the new classmate who is considered as a candidate for the team).^5^

Compound representations maximize contiguity. They can also be found in tasks that are used in developmental research. The marble tube task (Schlottmann, 2001), outlined in the Introduction, is such an example. Schlottmann (2001) found that even preschoolers utilized probabilistic information in the task. In discussing her results, she emphasized that performance of their participants revealed “intuitive” rather than analytic capabilities (see also Schlottmann & Wilkening, 2012, for insightful discussions). Given our findings, we should emphasize the role of task presentation. Under some presentation formats, intuition will yield a high level of accuracy, whereas under others it may fail. If the task is characterized by low contiguity and a high demand for selectivity, as in the Mouselab approach used by Betsch and colleagues (e.g., Betsch et al., 2014; Betsch, Lehmann, et al., 2018; Betsch, Lehmann, et al., 2016), intuitive capabilities do not suffice to solve the task properly. With this, our findings align with the basic tenets of default-interventionist models on the interplay of automatic and deliberate processes in decision-making (e.g., Evans, 2008; Glöckner & Betsch, 2008). The work of Söllner, Bröder, and Hilbig (2013) with adult participants has strikingly demonstrated that automatic integration, which enables a perception-like, holistic picture without mental effort, is only likely if information is accessible with minimal need for visual search. As soon as the presentation format requires visual search, automatic integration processes are impaired and deliberation becomes necessary (Söllner et al., 2013).

### Methodological and theoretical implications

The first lesson to be learned from our study pertains to the methodological level. Findings are contingent on research paradigms. To evaluate results, we have to take into account the relation between task properties (e.g., presentation format) and psychological processes. To compare tasks, it is helpful to analyze their properties within a conceptual framework. We need to *systematically control* and *vary task features* to assess capabilities in general and the level of decision competence in particular.

Second, sensitivity to probabilistic information under particular task conditions should not be confused with understanding. Conceptual understanding of the probability concept is not a necessary condition for utilization of probabilities as decision weights in a perceptual task. Children of the same age who solved a perceptual task with bravura might get lost if they face a structurally equivalent task under altered figural conditions. Our study shows that probability neglect within the same age group varies as a function of formally irrelevant changes in presentation. Due to randomization of participants to experimental conditions, these effects cannot be attributed to individual variations on the conceptual level. Conceptual understanding is a hypothetical construct that we cannot directly observe. In contrast, we can directly observe and measure performance (e.g., the portion of normatively correct choices). Performance, however, can result from many different processes, some driven by conceptual analysis and others by intuition. If we generally attributed good performance to understanding, we would neglect crucial differences in cognitive processes.

For illustration purposes, consider the following example. Mike and Tom separately go fishing for trout in different lakes and sell their catch at the market. Assume that each fish sells for the same amount of money (i.e., the value is a constant). Lakes, however, differ with regard to the prevalence of trout and so, accordingly, does the likelihood that fish are caught within a certain amount of time, say *p* = .7 for Lake A and *p* = .4 for Lake B. In time, Mike and Tom prefer Lake A. This performance is adaptive with regard to the probability distribution of the environment, assuming that the goal of two fishers is to maximize their gains at the fish market. A month later, Mike and Tom explore two other lakes, C and D. Again, there is the same strong difference with regard to the prevalence of trout, *p*_C_ = .7 and *p*_D_ = .4. However, there is an additional feature. Trout in Lake D frequently have highly distinct spotted patterns on their body. However, this is an irrelevant feature when it comes to selling, because they sell for the same price at the market as the unspotted ones. Nevertheless, we observe that Tom prefers Lake D containing spotted trout, whereas Mike prefers Lake C with the higher success rate in terms of catching fish. If we assume that the fishers’ aspirations have not changed (they still wish to maximize their profit at the market), the difference in performance is surprising and informative. It is surprising with regard to our initial impression that both had the same understanding of probability, given their convergingly adaptive behavior when choosing between Lakes A and B. It is informative, however, if we are interested in mental processes. The change in features of the environment appears to have affected Tom’s but not Mike’s behavior. One could hypothesize that they applied different rules when making their choices. When asked for the reasons behind their preferences, Mike might tell us that he precisely protocols information such as the location, number of fish caught, number of attempts, and time spent. Therefore, he knows exactly in which lake one catches more fish per hour. Tom, on the other hand, might tell us that he remembers very well in which lake he caught a certain fish. The last one had a pattern that reminded him of a smiley face. He never had a better catch than at the lake with the spotted trout. Mike’s strategy is surely analytic revealing an understanding of proportions.

Tom’s narrative is compatible with the availability heuristic described as an intuitive approach to judgment by Tversky and Kahneman (1973). According to this heuristic, one can judge probability or frequency by the ease with which instances come to mind. This heuristic exploits associative strength in memory, which is quite a valid proxy variable on many occasions because it reflects experienced frequencies. Yet availability can be biased if other features enhance the recall of events, such as the salient patterns on the bodies of trout from Lake D. In Lakes A and B, trout did not significantly differ in saliency. Accordingly, the ease with which exemplars can be recalled later should only be driven by the experienced frequency. In such an environment, application of the intuitive availability heuristic will result in a similar level of accuracy as formal rules. Most importantly, however, the availability heuristic can be applied without any understanding of the concepts of probability and chance. To distinguish concept-informed rules from intuitive heuristics, one must consider critical tasks in which the latter yields systematic biases. This research technique was the ingenious fundament of the heuristics-and-biases approach to the identification of mental processes (e.g., Kahneman, Slovic, & Tversky, 1982).

The example illustrates that adaptive performance should not be confused with conceptual understanding. The implication for research on cognitive development is straightforward. Environments that suit intuition are not overly informative if we wish to learn about the development of conception and understanding. For the sake of scientific progress, the detection of errors and failures is as important as demonstrating success in adaptation. This notion does not only apply to research with children. Conceptual understanding varies widely when it comes to probabilistic reasoning, even in adults. Base-rate neglect is a prominent example. Gigerenzer and Hoffrage (1995) showed that adults were able to use base-rates if probabilistic information was conveyed in a frequency rather than probability format. Granting this finding, one may be tempted to conclude that adult humans generally “understand” the basic principles of conditional probability theory if information were presented in a suitable format (i.e., in frequencies). Fiedler, Brinkmann, Betsch, and Wild (2000), however, demonstrated that even when probabilities were presented in frequency formats, adult participants were not immune to systematic biases. Their results indicate that these biases were due to a lack of understanding of the relation between probability, base-rate, and sampling constraints.

We showed that contiguity and selectivity are important features of a task. It is beyond the scope of this study, however, to uncover the processes associated with these different presentations. In line with Wohlwill’s (1968) approach, one should expect that moving along the continuum from perceptual to conceptual tasks, cognitive processes move from bottom-up to more top-down processing. Indicators for such a tendency should also be found in adults, since a number of aspects might encourage rapid bottom-up processing in perceptual tasks. The decrease of the demand for selectivity results in increased salience of the relevant information. In the presence of contiguity, it becomes less likely that the individual must actively engage in linking relevant information (e.g., weights, prediction values, options). These advantages might manifest themselves in behavioral processes such as differences in gaze patterns between the extreme conditions (high contiguity and low demand for selectivity vs. low contiguity and high demand for selectivity). Obviously, one should expect that the number of changes of fixations is lower in the perceptual than in the conceptual condition. Additionally, specific gaze pattern and fixation times might indicate differences in top-down driven elaborations. To uncover the processes associated with different presentation formats, it might be a promising line of further research to use eye-tracking methodology in this paradigm.^6^

### Evaluating decision competence in children

If sensitivity to probabilistic information under particular task conditions does not necessarily imply a probabilistic understanding, how can we then evaluate decision competence in children? To grasp the limitations in the competence of children in terms of using probabilities as decision weights, it may help to consider results from decision research with adults. Adults were found to make use of a so-called equal-weight rule, which ignores probabilities (i.e., weights each outcome with a constant instead of its stated probability; Payne et al., 1988). Application of this rule can be adaptive in environments in which the dispersion of probability is low. Accordingly, a smart decision maker with proper insight into the formal underpinnings of probability theory can deliberately decide to neglect a low-dispersive probability distribution, but at the same time use probabilities in a different environment.

Yet what are the necessary conditions for judging decision competence? To address this issue, we have to entangle the dynamics of stability and variation in decision behavior. An adaptive decision maker will change between strategies of searching and utilizing decision information depending on context features. This is a backbone assumption of the bounded rationality approach (Simon, 1955; see also Shah & Oppenheimer, 2008). It requires the decision maker to be highly sensitive to context features (e.g., weight distributions), effectively manage resources (e.g., limiting information acquisition under time pressure), and put decision-making under executive control (e.g., strictly prioritizing and focusing strong-weight information and suppressing irrelevant information). These capabilities together enable the individual to change decision behavior contingent upon contextual changes. The resulting behavioral variations are manifestations of decision competence. They must not be confused with within-task variations. Decision theory scaffolds this position by the axioms of rationality (e.g., von Neumann, & Morgenstern, 1947). One of them, the axiom of invariance, demands that decisions should be immune to variations in presentation if the structure and content of the task are unchanged. Obviously, children in our study violated this axiom. The rate with which children utilized probabilities differed greatly between presentation conditions even though the structure of the task and situation remained constant.

In a nutshell, a competent decision maker should show invariance in decision behavior irrespective of changes to the presentation format. However, he or she should adapt to variations in task structure and environmental demands (i.e., by changing strategies of information acquisition). In probabilistic environments, even 9-year-old to 10-year-old children exhibit severe shortcomings in adaptively tuning information search and decisions to the structure and environmental demands (e.g., Betsch, Lehmann et al., 2018; Betsch, Lehmann, et al., 2016; Lindow & Betsch, 2018; Mata et al., 2011). In addition, their performance strongly varies depending on presentational features—as the present research demonstrates. As such, the pronounced tendency to utilize probabilities under high contiguity and low demand for selectivity cannot be interpreted in isolation to the other conditions. Due to the fact that probability neglect increases in the counter conditions, the *differences between conditions* must be considered an indicator of decision competence. These differences highlight the deficit.

### The problem of conflict in choice tasks

In our study, the preschoolers’ success rate was around chance in some conditions and quite low across all manipulations, even in conditions with high contiguity. This finding is inconsistent with results from other studies showing that even children younger than 6 years are responsive to variations in probability in different domains, such as causal reasoning (Gopnik & Sobel, 2000), judging preference (Kushnir, Xu, & Wellman, 2010), trusting informants (Pasquini et al., 2007), and evaluative judgment (Schlottmann, 2001).

Note that we obtained these results although we encouraged children to utilize probabilistic information by instruction (i.e., animals will help, but their smartness matters). Moreover, we reinforced cue validities using payoff feedback, and only considered children in our analyses who passed the manipulation check before and after the test sessions (i.e., children who recognized which animal was the smartest in predicting treasures).

One might speculate that our paradigm was simply too demanding for young children to learn and use probabilistic information. Betsch, Lang, Lehmann, Förster, and Stelzel (2020) decomposed the treasure-hunt paradigm into three consecutive steps involving discrimination between probabilistic cues, making choices given the prediction of one probabilistic cue, and making choices given the predictions of two cues making contradictory predictions. The results showed that children (6 vs. 9-year-olds) performed equally well as adults in discrimination tasks and inferences based on one probabilistic cue. However, when two cues were present that made contradictory predictions, a strong age effect emerged. Six-year-olds failed to utilize probabilities to differentially weight predictions, whereas some 9-year-olds tended to do so, although they did not achieve adult-levels of performance. The latter finding is in line with the results from the present study showing that young children still have problems weighting probabilistic cues under conflicting predictions. This suggests that conflict in multiple-cue choice is responsible for drops in performance rather than overcharging probability learning in the current paradigm. Gualtieri, Buchsbaum, and Denison (2019) presented children (4–5 years) with base-rate and testimony information that conflicted in one condition. Their results showed that children were responsive to both kinds of probabilistic information (although they refrained from integrating them when making probabilistic inferences). This finding appears to challenge a conflict account. Yet consider another recent study by Betsch and colleagues (2020). The authors integrated the trust in informants’ paradigm with Mousekids. Interestingly, young children used the informants’ validities when generalizing trust to other domains, but not for choices. The authors attributed these differences in performance to the specific nature of choice tasks. Contrary to judgment tasks, behavioral choices involve opportunity costs. If one option is chosen, the other is rejected, and the actor loses potential gains of the nonchosen alternative. In choice tasks based on the predictions of cues, as in our paradigm, participants face a double conflict: the conflict between predictions of the cues and the conflict inherent in choice tasks (i.e., the opportunity cost problem). Most evidence in support of probability utilization in children stem either from judgment tasks (see above) or feedback-based choice tasks (without probabilistic cues; e.g., Kerr & Zelazo, 2004).

At the moment, we can only speculate that young children’s drop in performance originates from being confronted with a conflict on two levels, incoherent predictions of the cues, and a choice among two promising options. A potential reason might be that this double conflict increases the need for confidence. Research in the domain of adult decision-making shows that conflicting information can result in an increase in evidence accumulation in order to increase confidence (Lee & Cummins, 2004). In the case of this more extensive consideration, the structure of weights can be changed in order to achieve coherence (Betsch & Glöckner, 2010; Betsch, Ritter, Lang, & Lindow, 2016; Glöckner & Betsch, 2008). Consequently, the “neglect” of probabilities on the surface level might stem from a process of restructuring weights in order to increase coherence. Accordingly, future research on children’s decision-making might explore the link between subjective confidence under conflict and its effects on the maintenance of or changes in the subjective structure of weights.

## Supplementary Information

ESM 1 (SAV 10 kb)

## Footnotes

1 Cue validity is differently defined in the literature. Our approach reflects the conceptual background from research on decision-making in a multiple cue environment (e.g., Jekel, Glöckner, & Bröder, 2018).

2 This debate is not limited to developmental psychology but is also a debated issue in research on judgment and decision-making in adults (see, e.g., research on base-rate neglect, which we briefly examine in the discussion).

3 In a noncompensatory environment, dispersion of weights (probabilities or cue validities) is so high that it is formally not possible to compensate the weighted value of the HVC’s predictions by the sum of the weighted values from all other cues. In our environment with two options and two outcomes, the low-validity cue must be neglected because its predictions are exactly at chance. Corrected for chance level, the remaining two cues have a validity of .17 (.67–.50) and .33 (.83–.50). Thus, the second cue’s predictions can never outweigh the predictions of the HVC if they are weighted by their probabilities (see Jekel, Glöckner, Fiedler, & Bröder, 2012)

4 We thank an anonymous reviewer for making us aware of this possibility.

5 According to the results of our study, the probabilistic cue (jersey with the number 10) should be less likely to be used if it was spatially separated from the visual representation of the girl (i.e., the girl wears a neutral T-shirt and the jersey is placed on the chair next to her together with her school bag). Such a cue constellation converged with our low-contiguity condition in which the probabilistic cue was presented next to the option.

6 We are grateful to one anonymous reviewer who suggested this way of research.
